# The effect of subacute poisoning with fenpropathrin on mice kidney function and the level of interleukin 1β and tumor necrosis factor α

**DOI:** 10.1007/s11033-020-05480-w

**Published:** 2020-05-08

**Authors:** Maria Jaremek, Barbara Nieradko-Iwanicka

**Affiliations:** 1 Neuropsychiatric Hospital in Lublin, Abramowicka 2 Street, 20-442 Lublin, Poland; 2 Medical University of Lublin Chair and Department of Hygiene, Radziwillowska 11 Street, 20-080 Lublin, Poland

**Keywords:** Fenpropathrin, Interleukin 1β, Tumor necrosis factor

## Abstract

Fenpropathrin (FEN) is a pyrethroid insecticide. Mammals can be exposed to these compounds with food and water as non-target organisms. Pyrethroids are classified into two types depending on chemical structure and neurotoxic effects. FEN has features of Type I and Type II pyrethroids. There is data that pyrethroids apart from neurotoxic properties, can be also nephrotoxic and immunotoxic. The aim of the study was to assess the influence of FEN on kidney function and concentration of proinflammatory cytokines: tumor necrosis factor alpha (TNFα) and interleukin 1 beta (IL-1β) in mice kidneys. Sixteen female mice were randomly divided into two groups: I—receiving saline and II—receiving FEN at the dose of 11.9 mg/kg *ip* for 28 consecutive days. On day 29 blood samples were obtained to measure serum creatinine concentration. The animals were killed, and kidneys were obtained in order to measure TNFα and interleukin IL-1β in mice kidneys with use of ELISA assay. The concentration of creatinine was (mean ± SD) in controls 0.2 ± 0.0 mg/dl in the group exposed to FEN 0.225 ± 0.046 mg/dl. TNFα concentration in the kidneys of controls was 6.154 ± 1.597 pg/ml and in the group intoxicated with FEN it was 6.318 ± 1.012 pg/ml. IL-1β concentration in the kidneys of controls was 4.67 ± 1.154 pg/ml while in the group intoxicated with FEN 27.983 ± 26.382 pg/ml (p < 0.05). In conclusion: FEN slightly affects kidney function and increases the concentration of proinflammatory IL-1β in mice kidneys, which supports the hypothesis about nephrotoxic and immunotoxic effect of this insecticide in non target organisms.

## Introduction

Pyrethroids are synthetic insecticides. They are 2250 times more toxic to insects than to vertebrates because of lower body temperature of insects, more sensitive sodium channels, smaller body size and slower metabolism rate [1].

The main mode of action of pyrethroids is binding voltage-sensitive sodium channels in neurons and induction of prolonged depolarization in neurons [2]. Pyrethroids are classified into two types: I and II based on induction of either T syndrome (with tremor) or CS syndrome (with choreoatetosis and salivation) developing after intravenous or oral administration to rats at high doses [3, 4]. Breckenridge et al. have found that four α-cyano pyrethroids (λ-cyfluthrin, cypermethrin, deltamethrin and fenpropathrin—FEN) affected not only voltage-sensitive sodium channels, but also chloride channels in cell membranes [5]. Among vertebrates, the most sensitive to pyrethroids is fish [6]. Pyrethroids are commonly used in agriculture to increase crops, in household to control ants, flies, mosquitoes, cockroaches, termites, spiders, ants for protection of animals from ectoparasite insects and ticks, even for medicinal purposes to treat lice and scabies [7].

Humans as non-target organisms can be exposed to pyrethroids when spraying them in farming [8], when taking care of animals or using electrovaporizers to control household pests [9], with food and water [10].

Pyrethroids are widely used to protect from malaria and tick-borne diseases. The World Health Organization recommends pyrethroids (in particular deltamethrin, permethrin and α –cypermethrin) for in-home insect control [11]. The Center for Disease Control recommend pyrethroid repellents for pregnant women to protect against Zika virus infection [12].

However, there is a growing body of evidence that pyretroids are harmful to different organs and systems in mammals as they negatively affect fertility [13], immune system [14], cardiovascular system [15], may impair kidney and liver function [16], as well as glucose and lipid levels in the blood [17]. Their metabolites are excreted with urine [18] and to much lesser degree with feces.

FEN (α-cyano-3-phenoxybenzyl-2,2,3,3-tetramethylcyclopropanecarboxylate) has features of Type I and Type II pyrethroid. It is used in agriculture. It acts as a sodium and potassium channel blocker causing repetitive neuronal discharge [19].

The aim of the study was to assess the influence of FEN on kidney function and concentration of proinflammatory cytokines: tumor necrosis factor alpha (TNFα) and interleukin 1 beta (IL-1β) in mice kidneys.

## Materials and methods

The study project was approved by The Local Ethical Committee in Lublin, Poland (permission No 4/2009 dated 09.01.2009). The experiment was conducted according to Polish and European law regulations. Both authors had a training in planning and conducting experiments on animals. The experiment was conducted at the Center for Experimental Medicine at The Medical University of Lublin.

FEN was purchased form Organic Chemistry Institute (Annopol 6, 03-236 Warsaw). Saline was purchased from Glenmark Pharmaceuticals in ampules of 5 ml.

Sixteen female mice weighing 20–25 g were randomly divided into two groups: I—receiving saline and II—receiving FEN at the dose of 11.9 mg/kg of b.w. (suspended in saline) *ip* for 28 consecutive days. The dose was chosen because of our previous experience with FEN. On day 29 blood samples were obtained to measure serum creatinine concentration. Creatinine was measured with a kinetic method with ErbaMannheim XL-60 biochemistry analyzer. The kidneys were homogenized with a mechanical blender MPW-120 in 0.1 mol buffer of Tris–HCl, of pH 7.4. 0.5 g of kidney tissue was blended in 5 ml of buffer. The homogenates were centrifuged for 15 min (5000*g*) twice. The supernatant was used for measuring TNFα and IL-1β concentration with ELISA tests. The TNFα and IL-1β ELISA kits were purchased from manufacturer (Cloud-Clone Corp. USA, Houston, TX, USA).

The results were analyzed with IBM SPSS Statistics (v. 21).

## Results and discussion

In our study we have focused on kidney in the course of subacute poisoning with FEN, which is a commonly used insecticide as kidney is the organ which plays a major role in elimination of the xenobiotic and its’ metabolites [18].

To our knowledge it is the first study assessing the levels of proinflammatory cytokines in the kidneys of mice exposed to FEN.

The dose of 11.9 mg/kg FEN was chosen because of our previous experience showing that at the dose the pyrethroid reduces locomotor activity in mice and affects activities of superoxide dismutase and glutathione peroxidase in mice brains [20].

IL-1β is produced my macrophages, monocytes, endothelial cells, mesangial cells, Langerhans cells, chondrocytes, keratinocytes, lymphocytes T and B. The best described factor inducing synthesis of IL-1β is lipopolisaccharide present in cell walls of Gram- negative bacteria, but xenobiotics can also induce it. Anti-IL-1β drug anakinra is used for treatment of rheumatoid arthritis since 2001. It is also used for treatment of cryopyrin-associated periodic syndrome. The drug is tested in clinical trials for effectiveness in idiopathic juvenile arthritis and gout.

**Fig. 1:**
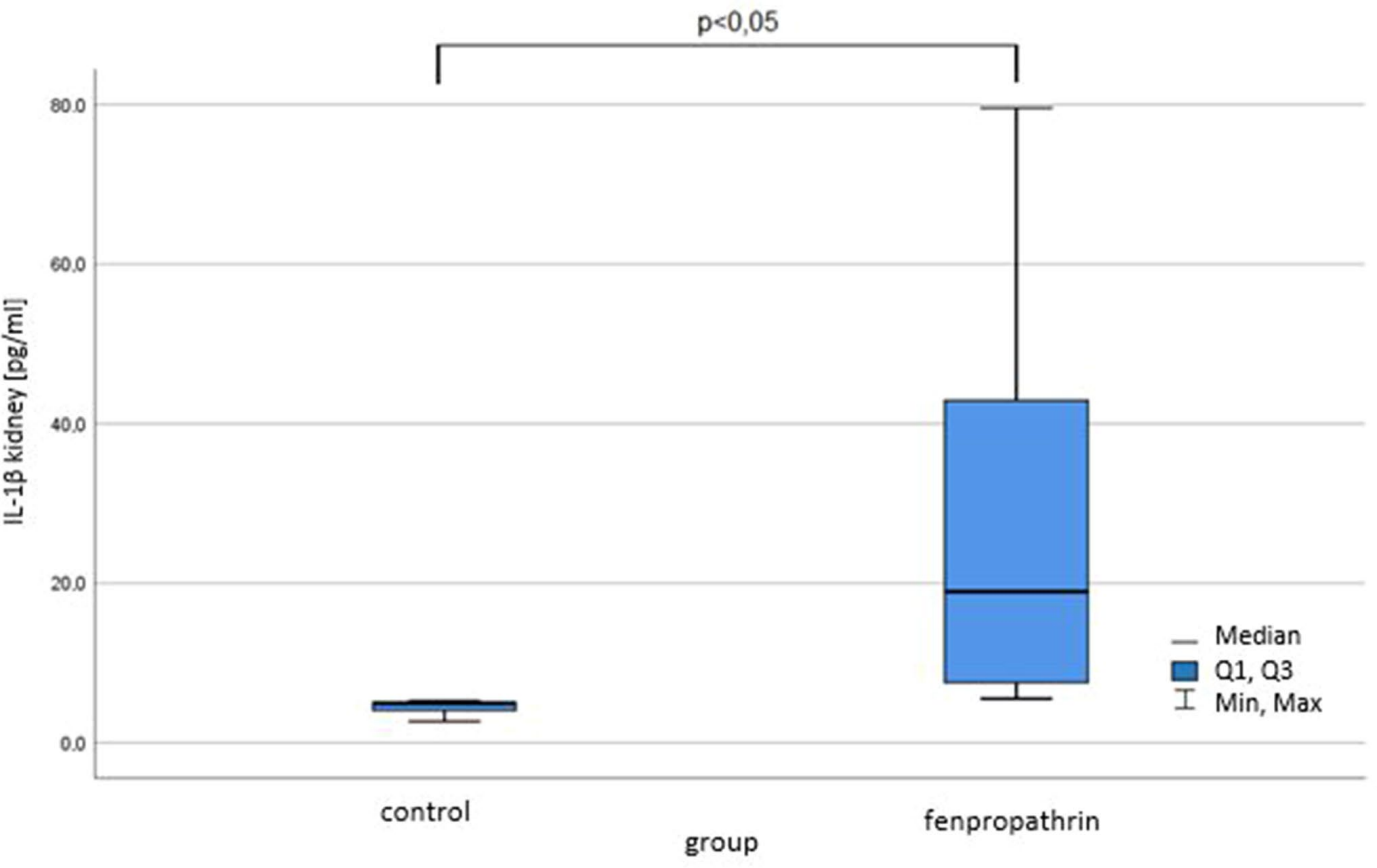
The influence of fenpropathrin on Il-1β concentration in mice kidneys; p < 0.05

**Fig. 2:**
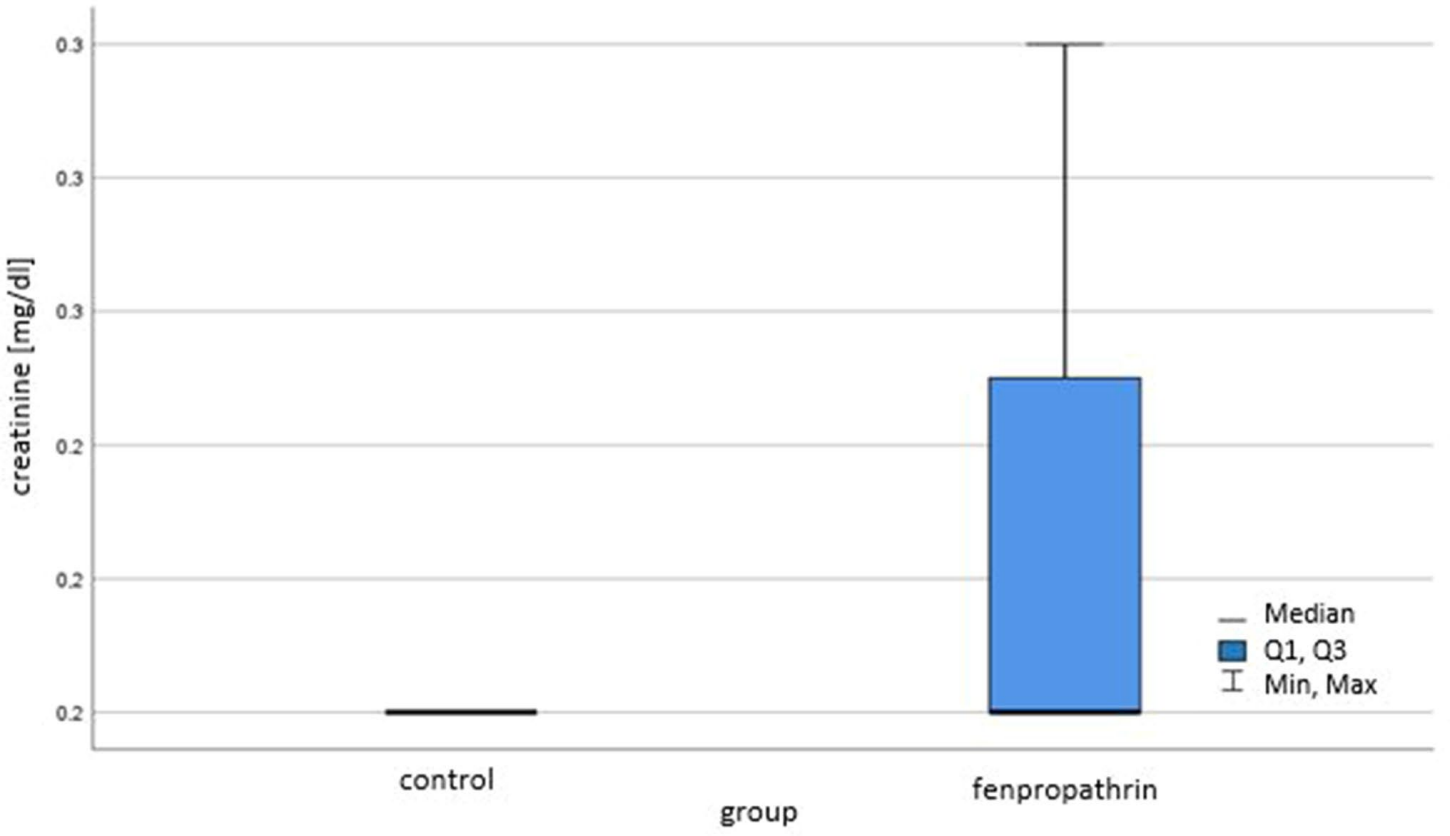
The influence of fenpropathrin on creatinine concentration in mice blood sera; p > 0.05

In our study IL-1β concentration in the kidneys of controls was 4.67 ± 1.154 pg/ml while in the group intoxicated with FEN 27.983 ± 26.382 pg/ml (p < 0.05), (Fig. 1). It suggests that due to subacute poisoning with FEN endothelial cells, mesangial cells and lymphocytes produce large amounts of the pro-inflammatory cytokine IL-1β even though there was no features of significand kidney dysfunction judging by serum creatinine concentration. The concentration of creatinine was (mean ± SD) in controls 0.2 ± 0.0 mg/dl in the group exposed to FEN 0.225 ± 0.046 mg/dl (Fig. 2).

TNFα plays an important role in immune and inflammatory response, regulates cell proliferation, excretion of immunomodulating substances and cell differentiation. TNFα is produced by lymphocytes T and B, macrophages, monocytes, fibroblasts, keratinocytes and neutrophils. In the XIX century Coley, a surgeon from New York, conducted a clinical study investigating the chances for remission of malignant tumor after bacterial infection. He manufactured Coley’s toxin, which was a supernatant of *Serratia mercescens* and *Streptococcus pyogenes* cultures. The Coley’s toxin was administered to 1200 patients with malignancies and in many a remission was recorded. It induced TNF production and high concentration of the cytokine. Apparently in some cases it induced cell death of neoplastic cells. Even though studies on Coley’s method of treatment were replaced by other anticancer therapies, many anti-TNFα drugs are used in connective tissue diseases’, psoriasis and Lesniowski-Crohn’s disease treatment: adalimumab, golimumab, certolizumab pegol, etanercept and infliksimab.

In our experiment TNFα concentration in the kidneys of controls was 6.154 ± 1.597 pg/ml and in the group intoxicated with FEN it was 6.318 ± 1.012 pg/ml. The difference between the two groups was not statistically significant. Considering not statistically significant change in serum creatinine concentration, FEN apparently does not produce cell death in the mice kidneys and probably in humans exposed to traces of the insecticide in food and water.

Type II pyrethroids can easily attach to receptors on the surface of lymphocytes T and B, therefore there is a discussion about their immunosuppressive action, risk of hypersensitivity induction and potential for use as adjuvants in antiviral and anticancer therapies [21].

## Conclusion

FEN slightly affects kidney function and increases the concentration of proinflammatory IL-1β in mice kidneys, which supports the hypothesis about nephrotoxic and immunotoxic effect of this insecticide on non target organisms.
